# The First-Ever Encounter with *Salmonella enterica* Serovar Hvittingfoss Causing Acute Gastroenteritis in India: A Case Report

**DOI:** 10.3390/idr16060094

**Published:** 2024-12-02

**Authors:** Mahadevaiah Neelambike Sumana, Morubagal Raghavendra Rao, Deepashree Rajshekar, Krishna Karthik, Nikita K Shah, Veerabhadra Swamy, Chinchana Shylaja Eshwar, Yogeesh D Maheshwarappa

**Affiliations:** Department of Microbiology, JSS Medical College and Hospital, JSS Academy of Higher Education and Research, Mysuru 570015, India; mnsumana@jssuni.edu.in (M.N.S.); morubagalrao@jssuni.edu.in (M.R.R.); drdeepu.rajshekar@gmail.com (D.R.); krishnakarthik46@gmail.com (K.K.); nikitakshah19@gmail.com (N.K.S.); veerabhadraswamygs@gmail.com (V.S.); chinchanaes@gmail.com (C.S.E.)

**Keywords:** non-typhoidal *Salmonella*, *Salmonella enterica* serovar Hvittingfoss, acute gastroenteritis, foodborne illness

## Abstract

**Background:** *Salmonella enterica* serovar Hvittingfoss, a member of the non-typhoidal *Salmonella* group, is an important foodborne serovar most frequently identified in regions (Australia, Belgium, and the United States) with active surveillance systems. This serovar has been implicated in outbreaks of foodborne illness. Soft cheese, crab cocktail, beef, and rock melon are commonly involved in these outbreaks. Although the definitive host of this serovar is not yet established, *Salmonella* Hvittingfoss can be found in wild animals (Feral pigs), bird populations (Bar-tailed godwit, Plumed whistling ducks, White-naped crane, and Sharp-tailed sandpiper), and domestic birds like chickens. **Case presentation:** We report the first case of *Salmonella enterica* serovar Hvittingfoss causing acute gastroenteritis in a 52-year-old male labourer and agriculturist from a rural area of Mysuru, South India. This is the first documented case of *Salmonella* Hvittingfoss causing acute gastroenteritis in India. **Conclusions:** While non-typhoidal *Salmonella* infections typically resolve on their own, they can be particularly dangerous for certain demographics, such as children, the elderly, and those with compromised immune systems. Therefore, studying the different serotypes of these infections in both clinical and subclinical cases is crucial for targeting effective surveillance, hygiene practises, and food safety measures that can mitigate their impact on public health.

## 1. Introduction

Salmonellosis is a disease of public health concern caused by bacteria of the genus *Salmonella* [1]. It is an important zoonotic pathogen that can cause infections in humans and domestic birds (Chickens) [2,3]. It is estimated that approximately 93.8 million cases of gastroenteritis are caused by *Salmonella* species, also one of the commonest causes leading to mortality associated with foodborne illness [3,4].

Salmonellosis is most commonly encountered by younger children residing in low- to middle-income countries (LMICs), with a great burden in Africa, Southeast Asia, and Eastern Mediterranean countries [3]. The most common serovars of *Salmonella* that cause human infections globally are *Salmonella enterica* serovar Enteritidis, *Salmonella enterica* serovar Typhimurium, and *Salmonella enterica* serovar Newport, accounting for 65%, 12%, and 4%, respectively [4].

*Salmonella enterica* serovar Hvittingfoss was responsible for a few outbreaks in the United States [5]. The most recent outbreak was recorded in 2016 and was caused by contaminated cantaloupes [6,7]. *Salmonella* Hvittingfoss was isolated from human, animal, and environmental sources, even in Northern Australia [8]. It was primarily isolated from wild birds like the Bar-tailed godwit (*Limosa lapponica*), Plumed whistling ducks (*Dendr cygna eytoni*), White-naped crane (*Antigone vipio*), and Sharp-tailed sandpiper (*Calidris acuminata*), and domestic birds like Chickens. Additionally, they are also found in reptiles and Feral pigs (*Sus scrofa*) [9]. As per the available literature, there is no documentation of *Salmonella enterica* serovar Hvittingfoss causing acute gastroenteritis in humans in this geographical region. Hence, it is noteworthy to report this case.

## 2. Case Presentation

A 52-year-old male labourer and agriculturist from a rural area of Mysuru, Southern Karnataka, presented to the hospital with complaints of lower abdominal pain associated with diarrhoea (6–8 episodes of loose stools per day). He did not have a known history of diabetes mellitus, hypertension, tuberculosis, or asthma. He had a history of travelling alone to Chamarajanagar 2 days prior and attending a community gathering, and none of his family members presented with similar complaints. A positive history of contact with cattle and poultry was noted.

Physical examination revealed a blood pressure of 130/70 mm Hg, a pulse rate of 88 bpm, and an oxygen saturation of 99% in room air. The patient had no icterus, cyanosis, clubbing, lymphadenopathy, or oedema. A per-abdominal examination revealed no organomegaly. Respiratory and cardiovascular system examinations were normal. The patient was conscious and oriented, had no focal neurological deficits, and was afebrile. On admission, a provisional diagnosis of acute gastroenteritis was made, and the patient was empirically started on an injection of ceftriaxone 2 g IV OD to cover all the bacterial pathogens causing gastroenteritis. Treatment was initiated after sending the stool sample for culture and sensitivity.

Relevant laboratory investigations were performed. Haematological parameters revealed a total leukocyte count of 10,650 cells/µL with a 92.8% predominance of neutrophils and lymphocytopenia (590 cells/µL). All other parameters were within normal limits. His serum electrolytes and liver function tests were normal. The HIV, HBV, and HCV statuses were negative. As the patient presented with abdominal pain, a urine sample was sent for culture to rule out a urinary tract infection. Both urine microscopy and culture results were non-significant.

Stool microscopy revealed moderate inflammatory cells. No RBCs or parasitic forms were seen. The sample was processed according to the department’s standard operating procedures, which concur with CLSI standards. Upon receipt of the sample, it was inoculated onto MacConkey agar and Hektoen enteric agar. The sample was also inoculated into Selenite F Broth, and a subculture was performed onto another Hektoen enteric agar plate after 2 h of incubation. The plates were incubated aerobically at 37 °C for 18–24 h. After the incubation period, MacConkey agar showed non-lactose fermenting colonies, while Hektoen and Selenite F sub-cultured Hektoen agar showed pale green colonies. The colonies underwent manual identification and antimicrobial susceptibility testing using biochemical reactions and an automated system (Vitek 2 Compact System—bioMérieux, France, using N-405 card).

The isolated produced cytochrome oxidase but indole, Christensen’s urease, and Simmons citrate was negative. Triple Sugar Iron agar showed an alkaline slant/acidic butt with plenty of H_2_S. The isolate produced plenty of H_2_S. Hence, the agglutination was carried out using group-specific antisera O4 or Hi, which showed no agglutination. The Vitek-2 system identified the isolate as belonging to the *Salmonella* group. The organism was found to be sensitive to ampicillin, ceftriaxone, trimethoprim/sulfamethoxazole, ciprofloxacin, and chloramphenicol and resistant to azithromycin as shown in Table 1. The isolate was sent to the ICMR–National Institute of Cholera and Enteric Diseases, a World Health Organisation collaborating centre for research and training on diarrhoeal diseases, to identify and confirm susceptibility patterns. The isolate was identified as *Salmonella enterica* serovar Hvittingfoss and was reportedly sensitive to ciprofloxacin, norfloxacin, ofloxacin, meropenem, trimethoprim/sulfamethoxazole, cefotaxime, chloramphenicol, and ceftazidime but resistant to azithromycin.

Once the diagnosis of gastroenteritis due to NTS was made, ceftriaxone was injected for 7 days. The patient improved symptomatically and was discharged after completing 7 days of the antibiotic course. While examining the psychosocial history to discover the possible source of infection, the patient reported no stress or mental health issues, indicating a stable psychosocial environment. He also reported being a vegetarian. The patient did not present with any hereditary conditions or genetic predispositions to bowel disease within the family and maintained proper personal and environmental hygiene. However, the patient had a history of contact with cattle and poultry, which could be a potential source of the infection.

## 3. Discussion

This case report serves as a reminder of the evolving landscape of infectious diseases and the need for vigilance in identifying and managing emerging pathogens such as *Salmonella* Hvittingfoss, which causes acute gastroenteritis in humans. In 1936, *Salmonella* Hvittingfoss was initially identified as the cause of acute gastroenteritis in Hvittingfoss, a village in Norway. Upon consumption of soft cheese, seven individuals from a single family experienced gastroenteritis. Isolates from stool cultures were identified as a novel serovar of *Salmonella*. It was suspected that the source of cheese contamination came from infected cows [10] in 1976, *Salmonella* Hvittingfoss was isolated from stool samples of six individuals in the Netherlands who had a history of group trips to Japan and Thailand. Among them, five individuals had symptoms of food poisoning, with a crab cocktail suspected as the probable source of contamination [5].

In 2010, 97 cases of *Salmonella* Hvittingfoss were confirmed among patrons of Subway restaurants across 28 counties in the state of Illinois. The outbreak resulted in the hospitalisation of at least 25 individuals due to severe illness. Despite extensive investigations, the specific food source responsible for the outbreak was never identified [11]. The European Union/European Economic Area (EU/EEA) identified 24 to 57 cases of *Salmonella* Hvittingfoss infection annually from 2013 to 2021, and the serovar was rarely detected from environmental sources [12] and wild animals, such as birds [13] and amphibians [14]. Interestingly, this serovar was rarely observed in Belgium (0–3 cases annually in the period 2013–2021) [15]. None of the previously discussed cases involving *Salmonella* Hvittingfoss reported symptoms beyond gastroenteritis.

In 2022, 6 out of 18 Chinese tourists who travelled to Hong Kong were admitted due to severe diarrhoea, and *Salmonella* Hvittingfoss was isolated from rectal swabs of these patients [16]. The probable source of transmission was the consumption of contaminated beef. The Korean Disease Control and Prevention Agency’s surveillance system, which monitors pathogens associated with acute gastroenteritis (AGE) among patients seeking medical care for symptoms such as diarrhoea and abdominal pain, focuses on identifying waterborne and foodborne diseases. According to their findings, among the *Salmonella* strains transmitted from other countries, *Salmonella* Anatum (12%) was the most frequently detected. Additionally, rare serovars such as *Salmonella* India, *Salmonella* Bovismorbificans, *Salmonella* Poona, *Salmonella* Wandsworth, and *Salmonella* Hvittingfoss were also identified in the surveillance data [16,17,18].

In most cases, serovar Hvittingfoss infection is confined to gastroenteritis. However, very recently, in 2023, this serovar was isolated from a 33-year-old Belgian woman, causing bacteremia and endometritis six days post-uterine aspiration in the context of a missed abortion. She had travelled to Indonesia two weeks before the positive blood and cervical culture [19]. Additionally, several cases of *Salmonella* Hvittingfoss causing pelvic abscesses have also been reported. Even though these cases are atypical presentations of *Salmonella* Hvittingfoss, it is important to remain vigilant as it can be responsible for sepsis or other complications.

As far as available records indicate, this case report marks the first instance of *Salmonella* Hvittingfoss being reported in India, specifically in the Mysuru region, with no prior documentation in this geographical area. Other serovars are also circulating in this region (Mysuru) of India. In 2019, a fatal case of *Salmonella enterica* serovar in Kentucky was reported from an immunocompetent patient, causing acute gastroenteritis and sepsis, which was fatal [20]. The same serovar was also reported in 2023 from the same geographical area, being ceftriaxone-resistant in a case of gastroenteritis [20].

This geographical area has also reported other serovars, highlighting that many serovars have yet to be identified. Although these non-typhoidal *Salmonella* infections are typically self-limiting, they may pose a serious threat to children, the elderly, and immunocompromised individuals. In this present case scenario, through detailed clinical history, we could establish that cattle and poultry could be potential sources of the serovar isolated. Hence, clinical history-taking becomes vital in establishing the source. Therefore, assessing the distribution of these serovars among clinical and subclinical cases may help reduce morbidity and mortality among vulnerable groups. Effective surveillance, hygiene practises, and food safety measures are crucial for prevention and control. Understanding their epidemiology aids in implementing targeted interventions to mitigate the impact on public health.

## 4. Conclusions

Our findings reveal the first documented case of *Salmonella* Hvittingfoss causing acute gastroenteritis in India. Non-typhoidal Salmonella serovars, including *Salmonella* Hvittingfoss, are established zoonotic pathogens. Meanwhile, human salmonellosis is commonly linked to consuming contaminated animal-derived foods, and contact with domestic and wild animals also poses a significant risk for *Salmonella* infection. The spectrum of illness caused by this pathogen ranges from mild to severe, presenting with symptoms such as fever, diarrhoea, and vomiting. Although salmonellosis typically resolves without medical intervention, it can pose serious risks to vulnerable populations such as infants, the elderly, and those with compromised immune status. Thus, comprehensive surveillance is imperative to understand this bacterium’s epidemiology including source identification, characteristics, and clinical manifestations. In this particular case, the potential source could be contact with poultry or cattle. Additionally, there is a lack of evidence in the literature for this serovar pointing towards a definitive source; hence, more epidemiological investigations need to be carried out in this regard.

## Figures and Tables

**Table 1:** Antimicrobial susceptibility pattern of *Salmonella enterica* serovar Hvittingfoss.

Antimicrobial Agent	Minimum Inhibitory Concentration (MIC)	Interpretation
Trimethoprim/Sulfamethoxazole	≥20	Sensitive
Amoxicillin/Clavulanic acid	≤2	Sensitive
Ampicillin	-	Sensitive
Cefixime	-	Sensitive
Ceftriaxone	≥0.25	Sensitive
Ciprofloxacin	≤0.06	Sensitive

## Data Availability

All data sets generated or analysed during this study are included in the manuscript.
